# Quantifying social semantics: An inclusive definition of socialness and ratings for 8388 English words

**DOI:** 10.3758/s13428-022-01810-x

**Published:** 2022-03-14

**Authors:** Veronica Diveica, Penny M. Pexman, Richard J. Binney

**Affiliations:** 1 School of Human and Behavioural Sciences, Bangor University, Gwynedd, Wales LL57 2AS UK; 2 Department of Psychology and Hotchkiss Brain Institute, University of Calgary, Calgary, AB Canada

**Keywords:** Word ratings, Lexical decisions, Semantic cognition, Social cognition, Grounded cognition

## Abstract

It has been proposed that social experience plays an important role in the grounding of concepts, and *socialness* has been proffered as a fundamental organisational principle underpinning semantic representation in the human brain. However, the empirical support for these hypotheses is limited by inconsistencies in the way socialness has been defined and measured. To further advance theory, the field must establish a clearer working definition, and research efforts could be facilitated by the availability of an extensive set of socialness ratings for individual concepts. Therefore, in the current work, we employed a novel and inclusive definition to test the extent to which socialness is reliably perceived as a broad construct, and we report socialness norms for over 8000 English words, including nouns, verbs, and adjectives. Our inclusive socialness measure shows good reliability and validity, and our analyses suggest that the socialness ratings capture aspects of word meaning which are distinct to those measured by other pertinent semantic constructs, including concreteness and emotional valence. Finally, in a series of regression analyses, we show for the first time that the socialness of a word's meaning explains unique variance in participant performance on lexical tasks. Our dataset of socialness norms has considerable item overlap with those used in both other lexical/semantic norms and in available behavioural mega-studies. They can help target testable predictions about brain and behaviour derived from multiple representation theories and neurobiological accounts of social semantics.

**Supplementary Information:**

The online version contains supplementary material available at 10.3758/s13428-022-01810-x.

## Introduction

Conceptual knowledge is the foundation of our complex interactions with the environment, bringing meaning to the objects, words, and social agents we encounter. ﻿A major challenge for the cognitive sciences is therefore to characterise how meaning is represented in the brain. Of particular interest has been the issue of how the mental representations of concepts become connected to their referents, termed the symbol grounding problem (Harnad, 1990; Searle, 1980). Within multiple representation accounts of semantic processing, concepts are mapped to the world, or *grounded*, by being directly represented within the neural systems underpinning multiple experiential channels such as perception, action, emotion, language and cognition (Borghi et al., 2018; Kiefer & Harpaintner, 2020). Sensorimotor systems are particularly important for grounding concrete concepts such as *festival* and *politician.* In contrast, abstract concepts like *romance* and *democracy* cannot, by definition, be directly experienced through the senses, and may thus rely to a greater degree on other types of information, such as affective (Fingerhut & Prinz, 2018; Kousta et al., 2011), introspective (Shea, 2018) and linguistic experience (Borghi et al., 2019; Dove, 2018). Further, there is growing recognition that there are different types of abstract concepts which depend to varying extents on these manifold sources of information (Harpaintner et al., 2018; Villani et al., 2019) and which elicit different patterns of behavioural responses in lexical-semantic tasks (Muraki et al., 2020).

Recently, there has been a rise in interest concerning the role that social experience plays in the acquisition and representation of concepts. Indeed, there are proposals in which social interaction and social context are pinpointed as a key source or mechanism for grounding that may be particularly important for the representation of abstract concepts (Barsalou, 2020; Borghi et al., 2019). For instance, Barsalou (2020) proposed that the social environment (e.g., agents, social interaction, culture) provides one form of grounding, in addition to that afforded by perceptual modalities, both of which are distinguished from the body, and the physical environment. Likewise, Borghi et al. (2019) argued that both social interactions and linguistic inputs are crucial for the acquisition of abstract concepts (also see Borghi & Binkofski, 2014). In Pexman et al. (2021), we have reviewed these theoretical perspectives as well as two parallel sets of empirical literature, which provide some evidence for socialness being a key principle underpinning semantic representation. For example, property generation and feature ratings studies found that social semantic content, or *socialness*, helps distinguish concrete from abstract concepts (Barsalou & Wiemer-Hastings, 2005; Troche et al., 2014; Wiemer-Hastings & Xu, 2005) and even different sub-types of abstract concepts (Harpaintner et al., 2018; Villani et al., 2019). In parallel, a set of neuroimaging studies have found that words high in socialness are associated with differential patterns of brain activation during semantic processing (e.g., Arioli et al., 2021a, b; Binney et al., 2016; Mellem et al., 2016; Rice et al., 2018; Wang et al., 2019; for another review, also see Conca et al., 2021). Some authors have argued for a special status of social concepts over other types of concept, and have suggested that socialness may even be a fundamental driver behind the functional organisation of the semantic system (Lin et al., 2018; Ross & Olson, 2010; Simmons et al., 2010; Zahn et al., 2007). These studies were all based on limited word samples, but they provide some evidence that social words might be a distinct type of concept, in line with proposals of some multimodal (e.g., Borghi et al., 2018) and neurobiological models (e.g., Olson et al., 2013) of conceptual processing.

These theories are nascent and there are many outstanding questions about the nature and extent of the contribution that socialness makes to semantic representation. One fundamental question is whether socialness is a behaviourally relevant principle as indexed, for example, by its ability to account for variance in performance on lexical-semantic tasks. However, the extant empirical support is limited by the way socialness has been defined and measured. To our knowledge, the largest source of openly available socialness norms was compiled by Troche et al. (2017) and includes social interaction ratings for 750 English nouns. Another dataset collected by Binder et al. (2016) includes ratings for 434 nouns, 62 verbs, and 39 adjectives on four socially relevant dimensions labelled social, communication, human and self. Thus, the scale and scope (i.e., the syntactic classes of words) at which socialness has been explored has been limited to date. Moreover, *socialness* as a construct has been defined variably in terms of behavioural descriptiveness, and there is no consensus on the criteria that differentiate social from non-social concepts. The heterogeneity in definitions is summarised by Pexman et al. (2021); some researchers have measured socialness as, for example, the degree to which a word’s meaning refers to relationships between people (Troche et al., 2014, 2017), to social as opposed to individual contexts (Arioli et al., 2021a), or to the relationship between self and others (Crutch et al., 2012), and socialness has also been defined as how well words describe social behaviour (Zahn et al., 2007). This variability in the operationalisation of socialness hinders our ability to compare findings across studies and glean a broader understanding of the contribution made by socialness to conceptual representation in the brain, and its behavioural consequences. Thus, we argue that to further progress theory, the field must first establish a clearer working definition of socialness.

Moreover, many of these past studies employed socialness definitions that emphasise specific aspects of social experience (Pexman et al., 2021). These narrow definitions might neglect important aspects of our highly complex interactions with the social environment. Thus, taking a crucial next step for understanding the construct of *socialness,* we aimed to collect ratings using an inclusive definition designed to capture all manner of features that are deemed to be socially relevant. This allowed us to test the extent to which socialness is reliably perceived as a broad construct. Relatedly, our socialness definition can be equally applied to a wide range of words, from nouns like those referring to social roles (e.g., *lawyer*) or institutions (e.g., *government*), to verbs like *to befriend*, and adjectives like *trustworthy*. This broad and inclusive definition can be used as a starting point for future studies exploring more fine-grained aspects of the socialness construct.

In summary, the aims of the present study were as follows: 1) collect socialness ratings for a large set of English words to provide a useful resource for future research endeavours; 2) use an inclusive definition to assess the extent to which socialness is reliably perceived as a broad construct; 3) explore to what extent these new socialness ratings capture aspects of word meaning that are distinct from those measured via other related semantic variables, such as concreteness and emotional valence, and 4) test whether socialness is a behaviourally relevant construct.

## Method

### Participants

Participants were recruited via the online platform Prolific (https://www.prolific.co/). Responders were restricted to those who self-reported being fluent in English and having no language disorders. A total of 605 participants (359 male, 240 female, six unspecified, *M*_age_ = 29.44 years, *SD*_age_ = 10.6) completed the study. Participants completed the rating task in 34 minutes on average and were compensated with GBP £4. Following exclusions (see below), the final sample consisted of 539 participants, with ages ranging from 18 to 76 years (*M* = 29.7; *SD* = 10.67). Of the participants, 216 (40.07%) were female, 317 (58.81%) male and six (1.11%) unspecified. English was the first language for 273 (50.65%) participants. Of the remaining 266 (49.35%) participants, 111 self-reported as being proficient in English, 124 advanced and 31 beginner/intermediate. A total of 185 (34.32%) participants were monolingual, while the remaining 354 (65.68%) reported speaking more than one language.

### Stimuli

The stimuli were 8948 words, including 5569 nouns, 1343 verbs, 2009 adjectives, and 26 other parts of speech (based on the dominant part-of-speech norms in Brysbaert et al., 2012) 1. We compiled our stimulus set from two sources: the Calgary Semantic Decision Project (Pexman et al., 2017) and Brysbaert et al. (2014)’s dataset of concreteness ratings. Ratings on emotion dimensions (valence, arousal, dominance) from Warriner et al. (2013) and on concreteness from Brysbaert et al. (2014) are available for all of the words included and the selected words span the entire continuum of these dimensions. In addition, we specifically selected these words so that there would be considerable overlap with behavioural mega-studies and other theoretically important psycholinguistic dimensions, some of which were used in analyses reported below, whereas others might be of interest in future research (e.g., Calgary Semantic Decision Project (Pexman et al., 2017), the Lancaster Sensorimotor Norms (Lynott et al., 2020), the Glasgow norms (Scott et al., 2019), word association norms (De Deyne et al., 2019), word prevalence norms (Brysbaert et al., 2018)).

We used 30 of the 8948 words as a set of control items which were to be presented to every participant and used during the data cleaning process (see below). These words were selected based on the ratings received in a pilot study (*N* = 36 participants) that was run to obtain an initial assessment of whether participants understand the task instructions and, in particular, the description of the inclusive socialness measure, and whether they provide reliable ratings (for a detailed description, see Section S1 of Supplementary Materials). Control words were selected to vary in the mean pilot socialness ratings, as well as in their concreteness (Brysbaert et al., 2014) and valence ratings (Warriner et al., 2013).

In addition to the 8948 words, we selected 12 practice words to be rated before the main ratings task so that participants could become familiar with the task requirements. We selected practice words that vary in concreteness (Brysbaert et al., 2014) and valence (Warriner et al., 2013), and that span the whole range of the social interaction dimension as measured by Troche et al. (2017) to ensure that participants practised both items with high and with low socialness ratings.

We used Qualtrics software (Qualtrics, 2020) to create two questionnaires for presentation to participants. To facilitate efficient Qualtrics processing, we divided the 8918 words into two lists of 4459 words from which each participant saw a random subset. These lists were equated for letter length, frequency (log subtitle frequency; Brysbaert & New, 2009), concreteness (Brysbaert et al., 2014) and valence (Warriner et al., 2013) to control for the probability of selecting words with different characteristics from each list. The control words were then added to both lists, resulting in two questionnaires each with 4489 words.

### Procedure

The word stimuli were presented using Qualtrics (2020) and linked to the Prolific online recruitment platform (www.prolific.co). Following the consent form, a demographics survey and instructions, participants rated the 12 practice words, then proceeded to rate the main set of items. Each participant rated 370 words randomly selected from one of the two item lists, plus the 30 control words. The control words were randomly intermixed with other items. The full instructions given to participants are presented in Section S2 of supplementary materials. In short, the participants were asked to rate the degree to which the words’ meaning has social relevance by describing or referring to the following:

*a social characteristic of a person or group of people, a social behaviour or interaction, a social role, a social space, a social institution or system, a social value or ideology, or any other socially relevant concept.*

Participants provided their answers using a seven-point Likert scale presented horizontally below each word. In addition, there was an “I don’t know the meaning of this word” option. There were 25 words presented per page. We collected data until we obtained at least 25 ratings per word.

### Data cleaning

In total, we collected 241,575 observations. The data cleaning pipeline involved sequentially implementing several techniques consistent with recommendations for identifying careless or insufficient effort responders (Curran, 2016) and computer-generated random responding (Dupuis et al., 2019), as well as other data cleaning procedures used in previous word norming studies (Brysbaert et al., 2014; Pexman et al., 2019; Warriner et al., 2013). First, we removed data from participants if they completed less than 33% of the ratings task (*n* = 0), responded with “I don’t know the meaning of this word” for more than 25% of items (*n* = 8) and provided the same rating for more than 25 words in a row (*n* = 17). Next, we examined each participant’s ratings of the 30 control words and generated correlations with the mean ratings of those words obtained in the pilot study. We removed data from 36 participants with a correlation coefficient less than .20. We then computed the correlation between each participant’s ratings and the mean ratings of all other participants. We deleted data from five participants with a correlation coefficient less than .10. Finally, if more than 15% of raters reported not knowing a particular word, we removed those words from the analyses reported below. This led to the exclusion of 560 words.

The final dataset was comprised of 8388 words and 202,841 observations, of which 3542 were “I don’t know the meaning of this word” responses. Not taking into account the control words rated by all participants, each word in the final dataset had 21.92 valid ratings on average (*SD* = 1.68), ranging from 15 to 27 ratings. Overall, 7703 (91.83%) words had at least 20 valid ratings.

### Data analysis overview

Data pre-processing, analysis and visualisation was accomplished using RStudio version 3.6.1 (RStudio Team, 2020). We first computed descriptive statistics for the socialness ratings and assessed their reliability. Then, to begin to explore the nature of the information captured by the socialness dimension and characterize its relationship with other pertinent psycholinguistic constructs, we computed the zero-order correlations between the mean socialness ratings and a variety of lexical and semantic properties of the words. Next, we conducted a series of hierarchical regression analyses to examine whether the socialness measure is related to behaviour in lexical tasks, using behavioural responses from the English Lexicon Project (ELP) lexical decision task (LDT; Balota et al., 2007) and the English Crowdsourcing Project (ECP) word knowledge task (Mandera et al., 2020). The LDT outcome variables quantify the speed and accuracy with which participants could distinguish between words and non-word letter strings. The ECP RT outcome variable measures the speed with which participants could recognize a word as known to them, while the percentage of participants reporting not knowing a word (henceforth proportion unknown) is a measure of word prevalence. We selected these tasks because they require only a fairly shallow level of semantic access (Muraki et al., 2020) and thus provide a conservative test of the relationship between this measure and lexical semantic processing. In addition, in both of these tasks, all word stimuli received the same behavioural response (“word” in the ELP LDT, or “I know that word” in the ECP) unlike, for instance, semantic decision tasks (e.g., Pexman et al., 2017) which involve different responses for different types of words. All predictor variables were mean-centred and we used reaction times standardized as z-scores because these reduce the influence of individual differences on overall processing speed (Faust et al., 1999).

## Results

### Descriptive statistics

**Fig. 1:**
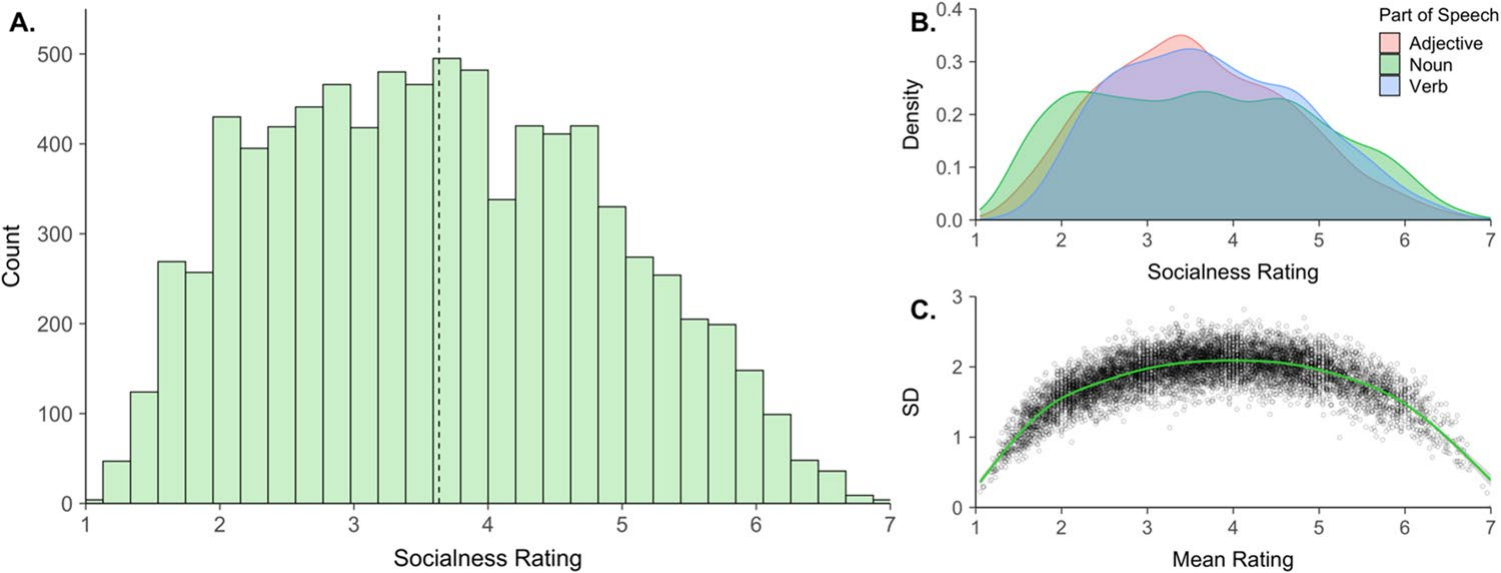
Distribution of socialness ratings. **A** Histogram of socialness ratings for 8388 words; the *dotted line* represents the mean. **B** Kernel density plot of ratings as a function of syntactic class. **C** Standard deviation of ratings plotted against their respective mean rating, along with a loess line (*in green*) that highlights the functional relationship

The raw data and resulting socialness ratings are provided on the Open Science Framework (OSF) project page (available at: https://osf.io/2dqnj/). The socialness ratings have a unimodal distribution with a mean of 3.63 (*SD* = 1.24) (Fig. 1a). More descriptive statistics for the mean ratings are provided in Table 1 and the distribution of ratings as a function of part of speech is depicted in Fig. 1b. The ratings have an average standard deviation of 1.85 (*SD* = 0.35) and participants provided more consistent responses at the extremes of the scale (Fig. 1c). Examples of words at the extremes of the socialness dimension are given in Table 2. Words like *friendship*, *people* and *sociable* received high socialness values, while words like *avalanche*, *millimeter* and *hemoglobin* received low socialness ratings, suggesting good face validity.

**Table 1:** Descriptive statistics for socialness ratings for 8388 words

Descriptive statistic	Value
Mean	3.63
Median	3.57
Standard Deviation	1.24
Minimum	1.05
Maximum	7.00
1st Quartile	2.62
3rd Quartile	4.58
Skewness	0.19
Kurtosis	– 0.80

**Table 2:** List of words at the extremes of the socialness dimension

Highest-rated words	Rating	Lowest-rated words	Rating
friendship	7.00	eucalyptus	1.05
socialize	7.00	horizontal	1.09
relationship	6.96	crocodile	1.09
people	6.90	sulfur	1.10
romance	6.78	sleeve	1.17
marriage	6.76	turbo	1.18
socialism	6.75	cranberry	1.18
political	6.73	dragonfly	1.18
family	6.72	hemoglobin	1.20
teamwork	6.72	shark	1.21
boyfriend	6.68	sunflower	1.21
friend	6.68	sandpaper	1.22
sociable	6.68	millimeter	1.22
sisterhood	6.67	avalanche	1.22
mother	6.67	spinach	1.22
democracy	6.65	airspeed	1.23
togetherness	6.65	button	1.23
sister	6.65	redwood	1.23
festival	6.64	pistachio	1.24
stepfather	6.64	birch	1.25
humankind	6.62	haystack	1.25
meeting	6.62	toothpaste	1.26
parental	6.62	paprika	1.27
befriend	6.61	cellophane	1.28
chatty	6.61	magnolia	1.28

### Reliability and validity

We first examined the reliability of the ratings by computing the one-way intra-class correlation coefficient (ICC) of all ratings using variances estimated via a random effects model with a global intercept and a random intercept per word (Brysbaert, 2019; Stevens & Brysbaert, 2016). We found an ICC of 0.9, which indicates good reliability of the mean socialness ratings. We further computed the split-half reliability for the 30 control words which were the only items in our dataset rated by all participants. We found a mean Spearman–Brown corrected split-half reliability of 0.998 (*SD* = 0.16) across 100 random splits, suggesting high reliability for the control items.

We then examined the validity of the ratings by computing the correlations between the ratings observed here and the mean ratings collected in the pilot study (*n* = 60 words), as well as two previous related sets of social interaction norms collected by Binder et al. (2016) (*n* = 258 words), and Troche et al. (2017) (*n* = 450 words). The current socialness ratings were strongly and positively correlated with the ratings collected in the pilot study (*r* = 0.97) and with the previous social interaction ratings collected by Binder et al. (2016) (*r* = 0.76) and Troche et al. (2017) (*r* = 0.76), suggesting good validity.

### Correlations with lexical and semantic properties

We examined the correlations between the socialness ratings and various lexical and semantic properties of the words. We included lexical dimensions in our analysis as previous work has shown that semantic content is not independent of the linguistic properties of words (Lewis & Frank, 2016; Reilly et al., 2012, 2017; Strik-Lievers et al., 2021). The lexical variables included letter length, orthographic Levenshtein distance (Yarkoni et al., 2008), phonological Levenshtein distance and frequency (log subtitle frequency; Brysbaert & New, 2009). To examine the proposed relationship between socialness and abstractness (Borghi et al., 2019), we included the following semantic variables that index sensorimotor experience: concreteness (the degree to which the word’s referent can be experienced through one of the five senses ; Brysbaert et al., 2014), imageability (the ease with which the word arouses a mental image ; Cortese & Fugett, 2004; Schock et al., 2012), body–object interaction (BOI; the ease with which a human body can physically interact with a word’s referent; Pexman et al., 2019), and sensory experience ratings (﻿the degree of sensory experience evoked; Juhasz & Yap, 2012). To assess the generalizability of the association between socialness and affective information reported in previous studies (Troche et al., 2014, 2017; Villani et al., 2019), we included in our analysis valence extremity (the degree to which the word evokes positive/negative feelings; this was measured as the absolute difference between the valence rating and the neutral point of the original valence scale by Warriner et al., 2013), arousal (the degree to which the word evokes feelings of arousal as opposed to calm; Warriner et al., 2013), and dominance (the degree to which the word evokes feelings of being controlled as opposed to in control; Warriner et al., 2013). Finally, to assess the relationship between the socialness ratings and linguistic experience, the semantic variables included semantic diversity (the extent to which a word appears in semantically diverse contexts; Hoffman et al., 2013), rating-based age of acquisition (AoA) (Kuperman et al., 2012), and a test-based AoA measure derived from (Dale & O’Rourke, 1981) and updated by (Brysbaert & Biemiller, 2017).

**Fig. 2:**
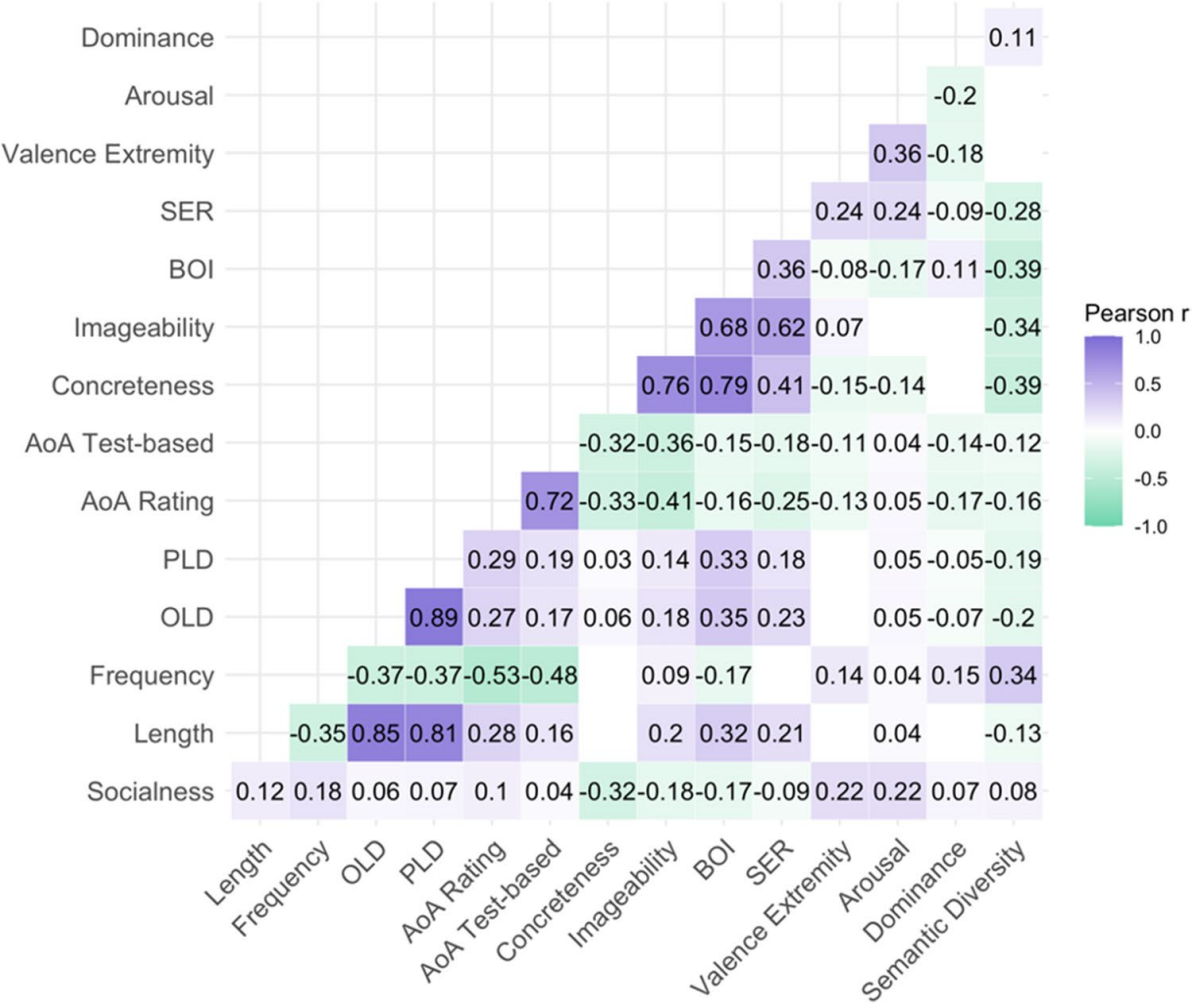
Correlations between mean socialness ratings and lexical-semantic dimensions. Only correlations significant at *p* < .01 are shown. The strength and direction of the correlation coefficients are indicated by the colour and the numerical values. For each variable of interest, the numbers of items in common with our socialness ratings are as follows: length, concreteness, valence, arousal, and dominance: 8388; log subtitle frequency: 8160; OLD and PLD: 8027; rating-based AoA: 8348; test-based AoA: 7321; imageability: 2680; BOI: 4038; SER: 2645. SER = sensory experience rating; BOI = body-object interaction; AoA = age of acquisition; PLD = phonologic Levenshtein distance; OLD = orthographic Levenshtein distance

These correlations revealed several interesting relationships that provide insight as to the nature of the word socialness measure (Fig. 2; see Fig. S1 for scatterplots). Socialness was negatively correlated with concreteness (*r* = – 0.32), imageability (*r* = – 0.18), and BOI (*r* = – 0.17), which suggests that words with less social relevance are associated with more embodied sensorimotor information. In contrast, socialness ratings were positively correlated with valence extremity (*r* = 0.22) and arousal (*r* = 0.22), suggesting that social words tend to have more affective information.

### Relationships with performance on lexical tasks

Next, we examined whether the socialness ratings are related to lexical-semantic processing using behavioural responses from the ELP LDT (Balota et al., 2007) and the ECP word knowledge task (Mandera et al., 2020). We conducted a series of item-wise hierarchical regression analyses in which we included other lexical and semantic predictors (that are typically related to behaviour in lexical tasks) in order to isolate the unique relationships of socialness to standardized reaction times (RTs), ELP error rates and ECP proportion unknown. In the first step, we entered the control predictors letter length, frequency (Brysbaert & New, 2009) and rating-based AoA (Kuperman et al., 2012). In the second step, we entered the semantic predictors: socialness, concreteness (Brysbaert et al., 2014), valence extremity (Warriner et al., 2013) and semantic diversity (Hoffman et al., 2013). We selected these other semantic predictors on the basis of multidimensional theories (e.g., Borghi et al., 2019) that highlight the simultaneous contribution of semantic variables derived from multiple sources, including linguistic (semantic diversity), sensorimotor (concreteness) and affective experience (valence extremity).

**Fig. 3:**
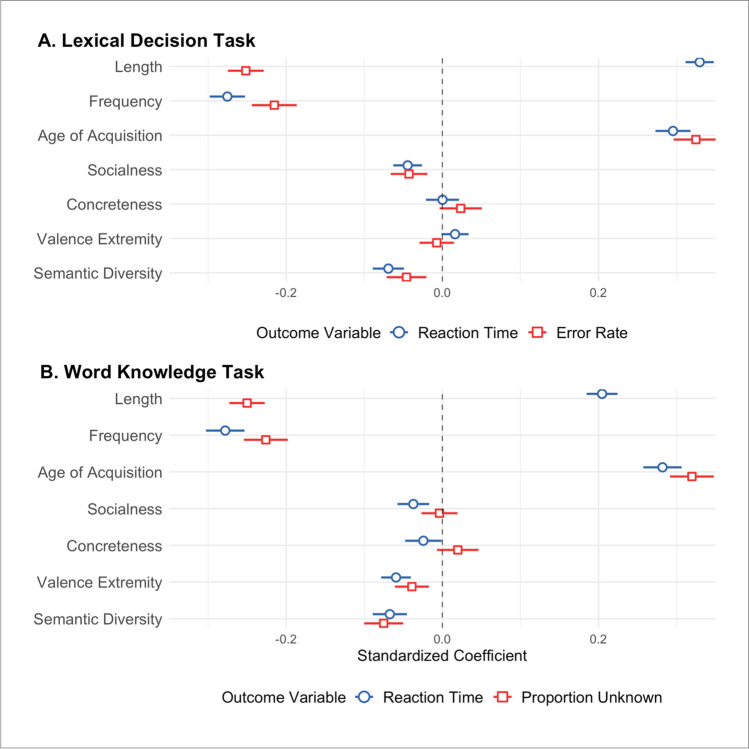
Standardized coefficient weights and 95% CIs for the second step of the hierarchical regression analyses predicting task outcome variables. **A** Standardized beta coefficients for LDT RTs (*blue*) and errors (*red*). **B** Standardized beta coefficients for ECP Word Knowledge Task RTs (*blue*) and the proportion of people reporting not knowing a word (*red*)

There were 6926 items for which we had values for all variables of interest in the analysis predicting LDT performance. Descriptive statistics and zero-order correlations between all variables of interest from this dataset are reported in Supplementary Table S1. The statistical results are reported in Table 3 and the standardized coefficients are illustrated in Fig. 3a. In this analysis, the control variables were all significant predictors of LDT latencies – RTs were faster for words that are shorter, more frequent and acquired earlier. There was significant improvement in model fit with the addition of the semantic variables, which collectively accounted for a further 0.61% of variance in LDT latencies. Of the semantic variables, only socialness and semantic diversity were significant predictors, with faster RTs for words with increased social relevance and for those encountered in more semantically diverse contexts. A similar pattern of results was observed when predicting LDT error rates. The control variables were all significant predictors, with fewer errors for words that are longer, more frequent and acquired earlier. There was significant improvement in model fit with the inclusion of the semantic variables, which accounted for an additional 0.56% of variance in LDT error rates. Socialness and semantic diversity were the only significant semantic predictors – error rates were lower for words with increased socialness and for those that are more semantically diverse.

**Table 3:** Regression coefficients from item-level analyses predicting lexical decision task latencies and error rates (*N* = 6926)

Predictor	zRTs							Error rates						
	*b*	*SE*	*t*	*p*	*sr*^*2*^	*R*^*2*^	*∆R*^*2*^	*b*	*SE*	*t*	*p*	*sr*^*2*^	*R*^*2*^	*∆R*^*2*^
Step 1						0.51							0.21	
Intercept	– 0.25	0.003	– 94.49	***				0.06	0.001	70.97	***			
Length	0.05	0.001	35.6	***	0.09			– 0.01	< .001	– 22.57	***	0.058		
Frequency	– 0.15	0.005	– 29.99	***	0.064			– 0.03	0.002	– 19	***	0.041		
Age of Acquisition	0.04	0.001	26.91	***	0.051			0.01	< .001	22.99	***	0.06		
Step 2						0.52	0.006						0.22	0.006
Intercept	– 0.25	0.003	– 95.06	***				0.06	0.001	71.21	***			
Length	0.05	0.001	35.75	***	0.089			– 0.01	< .001	– 21.5	***	0.052		
Frequency	– 0.13	0.005	– 23.9	***	0.04			– 0.03	0.002	– 14.7	***	0.024		
Age of Acquisition	0.04	0.001	25.78	***	0.046			0.01	0.001	22.31	***	0.056		
Socialness	– 0.01	0.002	– 4.73	***	0.002			– 0.003	0.001	– 3.57	***	0.001		
Concreteness	< .001	0.004	0.02	0.984	0			0.002	0.001	1.7	0.088	0		
Valence Extremity	0.01	0.004	1.83	0.067	0			– 0.001	0.001	– 0.64	0.525	0		
Semantic Diversity	– 0.07	0.01	– 6.77	***	0.003			– 0.01	0.003	– 3.54	***	0.001		

*Note.* b represents unstandardized regression weights. SE represents the standard error of the regression weights. sr^2^ represents the semi-partial correlation squared. zRTs standardized reaction times. *p < .05; **p < .01; ***p < .001

There were 7010 items for which we had values for all variables of interest in the analysis predicting performance in the ECP word knowledge task. Descriptive statistics and zero-order correlations between all variables of interest from this dataset are reported in Supplementary Table S2. The statistical results are reported in Table 4 and the standardized coefficients and illustrated in Fig. 3b. In this analysis, the control variables were all significant predictors of response latencies – RTs were faster for words that are shorter, more frequent and acquired earlier. There was significant improvement in model fit with the addition of the semantic variables, which accounted for a further 0.78% of variance in recognition RTs. All semantic variables were significant predictors, with faster RTs for words with increased socialness, concreteness and valence extremity and for those encountered in more semantically diverse contexts. The control variables were all significant predictors of the proportion of people reporting not knowing a word, with words that are longer, more frequent and acquired earlier being more prevalent. There was significant improvement in model fit with the inclusion of the semantic variables, which accounted for an additional 0.83% of variance in ECP proportion unknown. Valence and semantic diversity were the only significant semantic predictors – words that are more valenced and encountered in more semantically diverse contexts were reported as known by more people.

**Table 4:** Regression coefficients from item-level analyses predicting ECP word knowledge task latencies and proportion unknown (*N* = 7010)

Predictor	zRTs							Proportion unknown						
	*b*	*SE*	*t*	*p*	*sr*^*2*^	*R*^*2*^	*∆R*^*2*^	*b*	*SE*	*t*	*p*	*sr*^*2*^	*R*^*2*^	*∆R*^*2*^
Step 1						0.4							0.23	
Intercept	– 0.53	0.001	– 495.33	***				0.013	< .001	69.57	***			
Length	0.01	0.001	19.37	***	0.032			– 0.002	< .001	– 22.24	***	0.055		
Frequency	– 0.06	0.002	– 27.65	***	0.065			– 0.007	< .001	– 19.99	***	0.044		
Age of Acquisition	0.01	0.001	25.28	***	0.054			0.002	< .001	24.02	***	0.064		
Step 2						0.41	0.008						0.23	0.008
Intercept	– 0.53	0.001	– 498.44	***				0.013	< .001	69.93	***			
Length	0.01	0.001	20.21	***	0.034			– 0.002	< .001	– 21.67	***	0.051		
Frequency	– 0.05	0.002	– 22.07	***	0.041			– 0.006	< .001	– 15.74	***	0.027		
Age of Acquisition	0.01	0.001	22.5	***	0.043			0.002	< .001	22.38	***	0.055		
Socialness	– 0.003	0.001	– 3.6	***	0.001			< .001	< .001	– 0.31	0.754	0		
Concreteness	– 0.003	0.001	– 2.04	*	< .001			< .001	< .001	1.46	0.145	0		
Valence Extremity	– 0.01	0.001	– 6.09	***	0.003			– 0.001	< .001	– 3.52	***	0.001		
Semantic Diversity	– 0.02	0.004	– 6.01	***	0.003			– 0.004	0.001	– 5.89	***	0.004		

*Note.* b represents unstandardized regression weights. SE represents the standard error of the regression weights. sr^2^ represents the semi-partial correlation squared. zRTs standardized reaction times. *p < .05; **p < .01; ***p < .001

## Discussion

﻿Although some contemporary accounts (e.g., Barsalou, 2020;Borghi et al., 2019 ; Kiefer & Harpaintner, 2020) proffer a role for socialness in the organization and grounding of conceptual knowledge, many key questions remain about the nature of its contribution and its neural underpinnings. With the aim of facilitating future endeavours, in the present work we sought to 1) collect socialness norms for a large set of words; 2) test the extent to which socialness is reliably perceived as a broad construct; 3) explore to what extent socialness captures a distinct aspect of word meaning compared to those measured by other lexical and semantic variables, and 4) assess whether socialness can account for variance in behavioural responses in lexical tasks. To this end, we compiled the largest set of socialness norms available to date by collecting ratings for a set of 8388 English words, including nouns, verbs and adjectives. The socialness ratings show high reliability, and this suggests that the construct is meaningful to participants even at the broad and inclusive level of description provided. Moreover, the validity of the socialness construct was confirmed by a strong correlation with ratings on two other social semantic dimensions (Binder et al., 2016; Troche et al., 2017), despite the distinct definitions employed. However, our socialness measure shared around 58% of its variance with each of these other ratings, possibly reflecting differences in participant characteristics or perhaps methodological choices such as our more inclusive definition which might capture some additional aspects of social experience. Subsequent research will be needed to more thoroughly explore the precise aspects of our interactions with the social environment that are captured by this inclusive socialness measure, such as those measured by more restricted definitions (for examples, see Pexman et al., 2021).

Our preliminary analyses provide some important initial insights into the nature of the socialness dimension. First, while low socialness words tend to be concrete, high socialness words span the entire concreteness continuum, from concrete concepts like *mother*, to more abstract ones like *political*. In line with previous reports of a negative association between a social interaction measure and modality-specific perceptual ratings (Troche et al., 2017), we found that words high in socialness tend to be more abstract and to rely less on sensorimotor information. However, the present findings further suggest that socialness does not relate to concreteness in a simple linear fashion. Although theories of conceptual representation have proposed that social concepts are a sub-type of abstract concepts (Borghi et al., 2019; Kiefer & Harpaintner, 2020), this finding highlights the need to better understand the contribution made by socialness beyond this extreme of the concreteness dimension. Second, we found that words with increased socialness tend to be more valenced and arousing. This is in line with findings that social and affective dimensions reduce to the same latent factor of a multidimensional semantic space (Troche et al., 2014, 2017; Villani et al., 2019). Importantly, while the socialness ratings are significantly correlated with all the lexical and semantic variables explored here, the associated effect sizes are modest and suggest that the socialness measure captures a distinct aspect of word meaning. This is consistent with fMRI studies which found that the effect of socialness on brain activation during lexical-semantic processing is independent from that of a number of key semantic variables, namely imageability, concreteness, and valence, and suggest that socialness makes a unique contribution to semantic representation (Lin et al., 2018; Wang et al., 2019).

Using regression analyses, we have demonstrated for the first time that socialness of word meaning is related to performance in lexical tasks. This is true even at the broad and inclusive level of description provided. Specifically, we found a facilitatory effect on behavioural performance in lexical decision and word knowledge tasks – increased socialness was associated with faster decision latencies in both tasks and with better accuracy in the LDT. Importantly, this was true after controlling for other semantic variables known to influence lexical-semantic processing, namely concreteness, valence and semantic diversity. Further, this was true even in lexical tasks that involve only shallow semantic processing, where there is a limited pool of variance to be explained by semantic predictors. This unique contribution of the socialness measure suggests ﻿that it captures important information about semantic representation and processing and is in line with previous research on semantic richness effects. Semantic richness refers to the phenomenon whereby responses to words that are associated with relatively more semantic information tend to be facilitated in lexical and semantic tasks by virtue of their richer representations that allow faster and more accurate retrieval of meaning (for a review, see Pexman, 2012). ﻿As such, increased socialness might enrich a word’s conceptual representation and, consequently, facilitate lexical decisions via stronger feedback from semantic to orthographic representations (Hino et al., 2002; Hino & Lupker, 1996). Furthermore, our results suggest that socialness contributes to processing alongside other meaning dimensions derived from multiple experiential channels including linguistic (i.e., semantic diversity), sensorimotor (i.e., concreteness) and affective experience (i.e., valence). This is consistent with theories claiming that conceptual representation is multidimensional in nature and that social experience may be one of the underlying semantic dimensions (e.g., Borghi et al., 2019).

The ability of the semantic dimensions to explain variance in behavioural responses varied depending on the requirements of the task. While socialness and semantic diversity had a facilitatory effect on RTs in both tasks, concreteness and valence contributed to the word knowledge task, but not to the LDT. This is in line with research suggesting that conceptual representations are not stable across time and contexts; instead, the aspects of a word’s conceptual representation retrieved at any one point depend on the specific task/context (Pexman, 2020; Yee & Thompson-Schill, 2016). Our pattern of findings may be explained by the fact that LDT only requires the retrieval of some indication that a word has meaning, such as that indexed by its association with a multiplicity of meanings (i.e., semantic diversity). In comparison, the word recognition task might require access to additional features of a word’s meaning, like those that tap into the richness of associated sensorimotor (i.e., concreteness) and emotional experience (i.e., valence extremity). It might also suggest that socialness does not contribute additional semantic features to enrich a word’s conceptual representation, but is more indicative of the general relevance or salience of its meaning. This might be consistent with our finding that the socialness of a word does not account for variance in the number of people who know its meaning. Relatedly, it has been observed that social stimuli are preferentially processed during free viewing of complex naturalistic scenes, to the extent that socialness competes with the physical saliency of stimuli (End & Gamer, 2017, 2019). However, future research is needed to better understand the nature of the contribution made by socialness to the semantic richness of concepts (see Muraki et al., 2019 for an example of how to approach ﻿examining the factor structure of semantic richness). Moreover, it is important to highlight that, while the words we encounter are typically embedded in rich linguistic contexts (e.g., sentences) that shape our understanding of individual words, the socialness ratings were generated based on words presented in isolation. Future research should address this limitation by moving away from single word processing and considering the lexical-semantic properties of connected text/speech.

## Conclusions

In the present study, we compiled the largest set of openly available socialness norms to date. We used an inclusive definition, found that it produced reliable ratings and, thereby, showed that socialness has meaning as a broad construct. An important avenue for future research is identifying the specific aspects of social experience that are most related to conceptual processing to refine our working definition of socialness. Further, our explorations suggest that socialness captures an aspect of word meaning that is distinct to those measured by other key semantic variables and notably, an aspect of meaning that is behaviourally relevant. Our study also provides some initial insights into the information captured by the socialness measure, but subsequent work will be needed on this matter, as well as its role and behavioural consequences across the lifespan, including during acquisition, retrieval and when the semantic system is impaired. Thus, the socialness norms described here will enable future research into the organization and grounding of conceptual knowledge, and can help target testable predictions about brain and behaviour that can be derived from multiple representation theories (e.g., Borghi et al., 2019) and neurobiological accounts of social semantics (for an extensive discussion, see Pexman et al., 2021; also Binney et al., 2016; Binney & Ramsey, 2020; Diveica et al., 2021).

## Supplementary Information

ESM 1 (PDF 473 KB)

## Footnotes

1 Note that part-of-speech information was not available for one word: *hip hop*.
