# Supplementary material for: Quantifying social semantics: An inclusive definition of socialness and ratings for 8388 English words
Source: Behav Res Methods. 2022 Mar 14;55(2):461–73. doi: 10.3758/s13428-022-01810-x (PMC10027635; doi:10.3758/s13428-022-01810-x)
Supplement: Supplementary file 1 — (PDF 473 KB) [file 13428_2022_1810_MOESM1_ESM.pdf]

Supplementary Materials

for

Quantifying Social Semantics: An Inclusive Definition of Socialness and Ratings for 8,388 English Words

Veronica Diveica, Penny M. Pexman, Richard J. Binney

Contents:

- **Section S1.** Pilot Study p.2
- **Section S2.** Rating Task Instructions p.4
- **Section S3.** Additional Results p.7

Section S1. Pilot Study

1.1. Participants

Before starting the main experiment, we tested our socialness rating task in a sample of 36 participants (23 female, 13 male; M_{age} = 22.94 years, SD_{age} = 6.5). Participants were recruited from the participant pool at Bangor University. Participants completed the rating task in 24 minutes on average and were compensated with course credit. Of the participants, 17 saw version 1 of the instructions and 19 saw version 2.

1.2. Materials

We selected 60 items (including nouns, adjectives and verbs) that span the following dimensions: valence (Warriner et al., 2013), concreteness (Brysbaert et al., 2014) and social interaction (Binder et al., 2016; Troche et al., 2017). We created two versions of the instructions to assess whether wording influenced participants' understanding of the instructions and their ratings. In version 1, socialness was defined as the degree to which a word's meaning has a social quality, whereas in version 2 it was defined as the degree to which a word's meaning has social relevance. The rest of the instructions and examples were identical in the two versions.

1.3. Procedure

The word stimuli were presented using Qualtrics and linked to the online participant recruitment platform at Bangor University. Following the consent form, demographics survey and instructions, participants rated how well they understood the instructions on a 5-point Likert scale from not at all (1) to extremely well (5). Then, participants proceeded to rate all the items using a 7-point Likert scale presented horizontally below each word. In addition, there was an "I don't know the meaning of this word" option. Items were presented in random order and only one word was presented per page. Following 30% of the items,

participants were asked to explain the reasoning behind their chosen rating for the respective word. At the end of the ratings task, participants were asked to explain the task instructions using a text box.

1.4. Results

1.4.1. Understanding of Instructions

The data and analysis scripts can be accessed via the OSF project page (https://osf.io/2dqnj/). 8.33% of the pilot participant sample reported understanding the instructions moderately well, 55.56% very well and 36.11% extremely well. Most participants provided explanations that were consistent with our inclusive socialness definition (e.g.: "*Socialness describes anything related to people, for instance their interactions, ways of describing people, relationships, social places/event, social beliefs, etc.*"; "*relation to people and society*"). Interestingly, many participants focused their explanations on the link between the word's meaning and social interactions (e.g., "*Of, or relating to, the interaction of individuals. That which pertains to people interacting.*"; "*The socialness referred to how likely it was that the words were associated with social concepts by involving social interactions, by influencing social interactions, and by representing those and the values of those in social encounters.*").

1.4.2. Reliability

We examined the reliability of the ratings by computing the split half reliability for the 60 words. We found a mean Spearman-Brown corrected split-half reliability of 0.97 (*SD* = 0.12) across 100 random splits, suggesting high reliability. In addition, we assessed inter-rater reliability by computing the two-way random-effects intra-class correlation coefficient (ICC) based on absolute agreement. We found an ICC(2,1) = 0.4, 95%CI [0.33, 0.48] suggesting poor to moderate reliability of individual ratings and an ICC(2, 36) = 0.96, 95%CI [0.95, 0.97] suggesting excellent reliability of the average ratings across 36 raters. Moreover,

we found an ICC of 0.94, 95%CI [0.92, 0.96] which suggested that the 19 raters who saw the instructions eventually used in the main experiment (version 2) provided average ratings that were highly reliable.

1.4.3. The Influence of Instruction Version

A Pearson's Chi-square test of independence suggested that self-reported understanding of the instructions did not depend on the version of the instructions χ^2 (2, N = 36) = 0.92, p = 0.63. There was a strong positive correlation between mean socialness scores for the two instruction versions (r = 0.93, p <.001, R^2 = 0.87). Moreover, the reliability was comparable for the two versions, with a mean Spearman-Brown corrected split-half reliability of 0.93 (SD = 0.15) for version 1 and 0.95 (SD = 0.12) for version 2 (across 100 random splits). Therefore, we concluded that the wording did not significantly influence raters' responses.

Section S2. Rating Task Instructions

Our society and interactions with other people feature at the heart of many of our experiences in life and this study explores whether this is reflected in the language we use.

In particular, this study is interested in the 'socialness' of words, or the degree to which words can be considered 'social'. There are many ways in which a word can be considered 'social'. For example, a word is considered to be 'social' if it describes or refers to a social characteristic of a person or group of people (e.g., 'trustworthy'), a social behaviour or interaction (e.g., ' to fight'), a social role (e.g., 'teacher'), a social space (e.g., 'pub'), a social institution (e.g., 'hospital') or system (e.g., 'nation'), a social value (e.g., 'righteousness') or

ideology (e.g., 'feminism'), or any other socially-relevant concept. In contrast, 'non-social' words have meanings that lack in social relevance (e.g., 'chair', 'time').

Words also differ in the degree to which they can be considered social. Some words clearly refer to social things, social qualities or to social actions or events (e.g., 'trustworthy') whereas, for other words, the relationship to society or social interactions might only become apparent after a period of intense thought (e.g., 'promotion'), or not at all (e.g., 'chair'). The purpose of this study is to rate words based on the degree to which they have a meaning that has social relevance. Any word that in your estimation refers to something that has clear social relevance should be given a **high 'socialness' rating** (at the upper end of the numerical scale). Any word that in your estimation refers to something lacking in social relevance should be given a **low 'socialness' rating** (at the lower end of the scale). Any word that in your estimation refers to something that is not fundamentally social but has some social elements (e.g., 'smartphone'), or can be thought of as social in some circumstances (e.g., 'event'), should be given an intermediate socialness rating.

It is important that you base these ratings on the degree of the social relevance of the word's meaning and not whether the meaning is prosocial versus antisocial or evokes positive/negative associations. For example, the word 'fight' should be given a high 'socialness' rating because it refers to a type of interaction between people, and even though the interaction is antisocial.

Please make your 'socialness' ratings using the 7-point scale. A value of 1 indicates a low 'socialness' rating, and a value of 7 indicates a high 'socialness' rating. Values of 2 to 6 indicate intermediate ratings. Please feel free to use the whole range of values provided when

making your ratings. Click on the rating that is most appropriate for each word. When making your ratings, try to be as accurate as possible, but do not spend too much time on any one word. If you are not familiar with a word's meaning, please select 'I don't know the meaning of this word'.

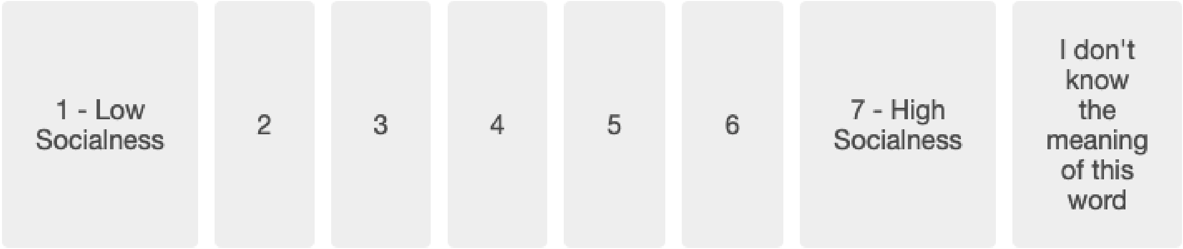

Section S3. Additional Results

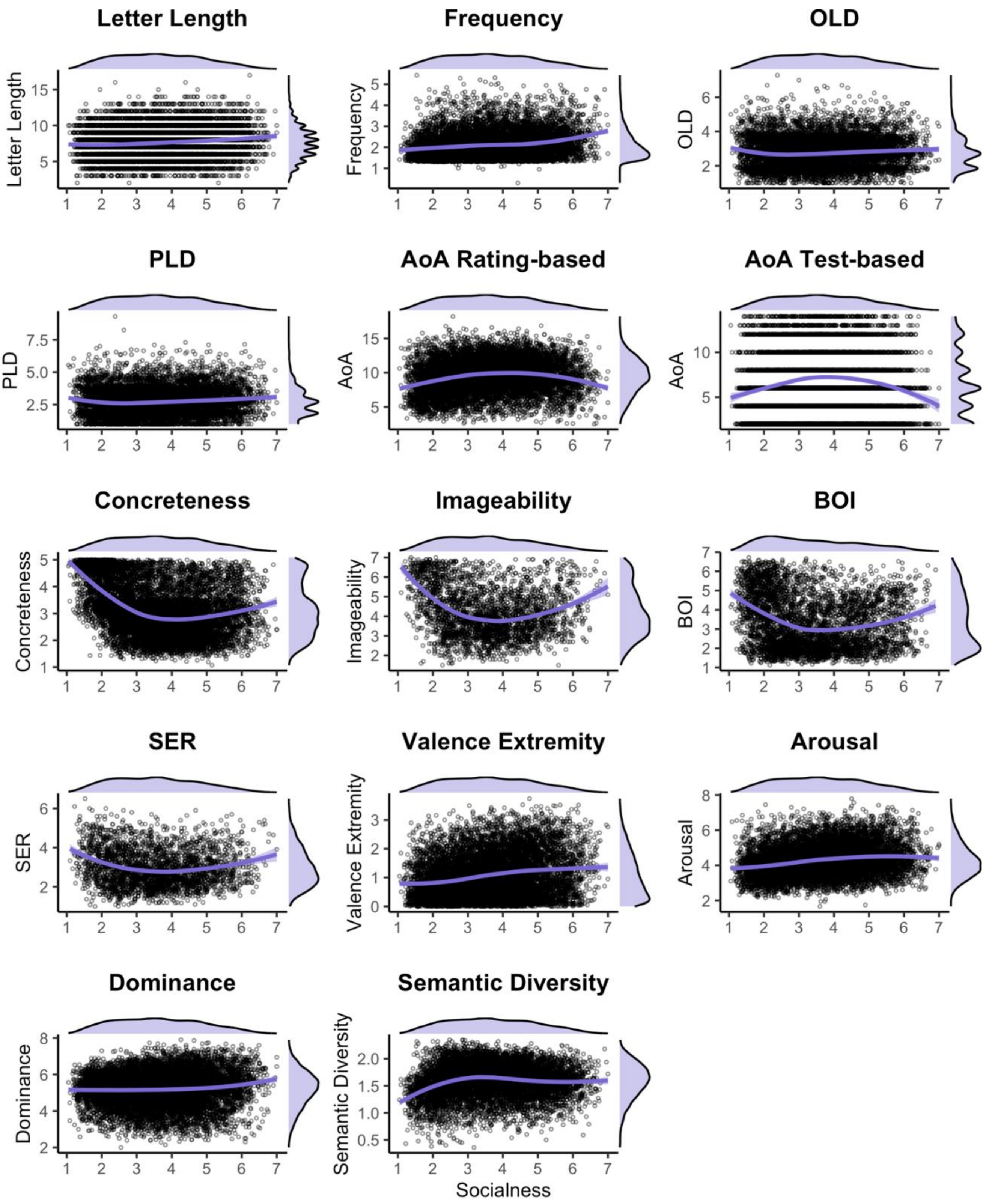

Figure S1. Scatterplots illustrate the relationships (highlighted by the loess line) between mean socialness ratings (x axis) and lexical-semantic dimensions (y axis). The density distributions of the socialness and variables of interest are plotted on the top and right

of the graphs respectively. For each variable of interest, the numbers of items in common with our socialness ratings are as follows: length, concreteness, valence, arousal and dominance: 8,388; log subtitle frequency: 8,160; OLD and PLD: 8,027; rating-based AoA: 8,348; test-based AoA: 7, 321; imageability: 2,680; BOI: 4,038; SER: 2,645. SER = sensory experience rating; BOI = body-object interaction; AoA = age of acquisition; PLD = phonologic Levenshtein distance; OLD = orthographic Levenshtein distance.

Table S1. *Means, standard deviations and correlations of all variables of interest for the regression analysis predicting performance in the English Lexicon Project Lexical Decision Task (N = 6,926)*

Variable	M	SD	1	2	3	4	5	6	7	8
1. Length	7.54	2.13								
2. Frequency	2.18	0.65	-.36**							
3. Age of Acquisition	9.43	2.44	.31**	-.57**						
4. Socialness	3.67	1.24	.13**	.18**	.09**					
5. Concreteness	3.08	0.95	-.06**	.07**	-.35**	-.29**				
6. Valence Extremity	1.07	0.77	.01	.14**	-.15**	.23**	-.14**			
7. Semantic Diversity	1.60	0.31	-.13**	.34**	-.16**	.09**	-.40**	.03*		
8. LDT zRT	-0.25	0.31	.52**	-.59**	.56**	-.03*	-.11**	-.08**	-.25**	
9. LDT Error Rate	0.06	0.08	-.08**	-.33**	.37**	-.10**	-.06**	-.10**	-.15**	.51**

Note. M and SD are used to represent mean and standard deviation, respectively. LDT = lexical decision task; zRT = standardized reaction times. * indicates p < .05. ** indicates p < .01.

Table S2. *Means, standard deviations and correlations of all variables of interest for the regression analysis predicting performance in the English Crowdsourcing Project Word Knowledge Task (N = 7,010)*

Variable	M	SD	1	2	3	4	5	6	7	8
1. Length	7.55	2.13								
2. Frequency	2.17	0.65	-.36**							
3. Age of Acquisition	9.43	2.43	.31**	-.57**						
4. Socialness	3.67	1.24	.13**	.18**	.09**					
5. Concreteness	3.08	0.95	-.06**	.07**	-.35**	-.29**				
6. Valence Extremity	1.07	0.77	.01	.14**	-.14**	.23**	-.14**			
7. Semantic Diversity	1.59	0.31	-.13**	.34**	-.16**	.09**	-.40**	.03*		
8. Recognition zRT	-0.53	0.12	.39**	-.55**	.53**	-.05**	-.11**	-.14**	-.23**	
9. Proportion Unknown	0.01	0.02	-.06**	-.35**	.38**	-.07**	-.06**	-.13**	-.18**	.63**

Note. M and SD are used to represent mean and standard deviation, respectively. zRT = standardized reaction times. * indicates $p < .05$. ** indicates $p < .01$.
